# *Arabidopsis* Qc-SNARE genes *BET11* and *BET12* are required for fertility and pollen tube elongation

**DOI:** 10.1186/s40529-015-0102-x

**Published:** 2015-09-02

**Authors:** Pablo Bolaños-Villegas, Cian-Ling Guo, Guang-Yuh Jauh

**Affiliations:** 1 Fabio Baudrit Agricultural Experimental Station, University of Costa Rica, La Garita de Alajuela, P.O. Box 183-4050, Alajuela, Costa Rica; 2 Institute of Plant and Microbial Biology, Academia Sinica, 128 Sec. 2, Academia Rd, Nankang, Taipei, 11529 Taiwan

**Keywords:** *Arabidopsis thaliana*, SNAREs, Fertility, Pollen tube elongation

## Abstract

**Key message:**

*BET11* and *12* are required for pollen tube elongation.

**Abstract:**

Pollen tubes are rapidly growing specialized structures that elongate in a polar manner. They play a crucial role in the delivery of sperm cells through the stylar tissues of the flower and into the embryo sac, where the sperm cells are released to fuse with the egg cell and the central cell to give rise to the embryo and the endosperm. Polar growth at the pollen tube tip is believed to result from secretion of materials by membrane trafficking mechanisms. In this study, we report the functional characterization of Arabidopsis *BET11* and *BET12,* two genes that may code for Qc-SNAREs (soluble *N*-ethylmaleimide-sensitive factor attachment protein receptors). Double mutants (*bet11*/*bet12*) in a homozygous/heterozygous background showed reduced transmission of the mutant alleles, reduced fertilization of seeds, defective embryo development, reduced pollen tube lengths and formation of secondary pollen tubes. Both *BET11* and *BET12* are required for fertility and development of pollen tubes in *Arabidopsis*. More experiments are required to dissect the mechanisms involved.

**Electronic supplementary material:**

The online version of this article (doi:10.1186/s40529-015-0102-x) contains supplementary material, which is available to authorized users.

## Background

Unlike in animals, sperm cells from flowering plants are non-motile and need to be delivered to the ovule by means of a pollen tube (Kawashima and Berger 2011). Once delivered, one sperm fuses with the egg cell to result in the diploid zygote and the other fuses with the secondary nucleus to generate the triploid endosperm (Berger et al. 2008; Kawashima and Berger 2011).

For the pollen tube to perform this task, it needs to elongate in a polar way. This growth mechanism requires highly coordinated interactions between the secretory machinery, the cell wall biosynthetic machinery and the cytoskeleton (Domozych et al. 2013). For instance, newly synthesized proteins first associate with the endoplasmic reticulum (ER), then are transported to the appropriate subcellular compartment (El-Kasmi et al. 2011). This process involves the fusion of ER-derived vesicles with the cis-Golgi cisternae, followed by protein sorting to the plasma membrane or lysosomes at the trans-Golgi network (TGN), a compartment also involved in endocytosis (El-Kasmi et al. 2011; Morita and Shimada 2014). The TGN performs two main functions: it sorts cargo destined for the plasma membrane and endosomes and receives cargo from endosomal compartments (Uemura and Nakano 2013).

Several proteins take part in membrane fusion events that occur in tip-growing plant cells. Examples are Rab-GTPases, which regulate the membrane recruitment and activity of tethering factors; Rab effectors such as phosphatidylinositol kinases; ATP-driven chaperones from the Sec1/Munc18 family, also called SM proteins; and soluble *N*-ethylmaleimide-sensitive factor attachment protein receptors (SNAREs) (Alpadi et al. 2012; Guo and McCubbin 2012; Karnik et al. 2013; Ohya et al. 2009). SNAREs are believed to be the principal determinants of membrane fusion specificity (De Benedictis et al. 2013). These proteins, 150-300 amino acids long, feature a membrane-anchored C-terminus, α-helical heptad repeats, and a cytosolic SNARE motif that functions during membrane fusion (Guo and McCubbin 2012; Uemura et al. 2012). A fusogenic SNARE complex comprises four SNAREs, including three with a central glutamine (Gln, Q) residue within the SNARE motif (the Q SNAREs), classified as Qa-, Qb-, and Qc-, and the R-SNARE, which features a central arginine residue (Arg, R) (Fujiwara et al. 2014; Uemura et al. 2012). In these complexes, the Q-SNAREs usually reside on the target membrane, whereas the R-SNARE resides on the vesicle membrane (Karnik et al. 2013). *trans*-QabcR–SNARE complexes are believed to function in individual intracellular fusion steps (Furukawa and Mima 2014), their topology varies in stringency (Alpadi et al. 2012), and formation of a fusion-competent membrane microdomain requires the presence of specific lipids such as sterols, diacylglycerol, and phosphoinositides (Furukawa and Mima 2014).

*Arabidopsis* has about 60 different genes in six groups that encode SNARE proteins (Lipka et al. 2007); most are members of protein families with more than one gene (Sanderfoot 2007). This large number of genes may be associated with the need in higher plants for polarized secretion (Sanderfoot 2007). Nonetheless, despite the sheer number of genes, most Q-SNAREs, including the Qa-SNAREs, show partial functional redundancy (Fujiwara et al. 2014; Morita and Shimada 2014). Examples are the *Arabidopsis* Qa-SNARE genes *SYP41,* *SYP42*, and *SYP43*, which are involved in secretory and vacuolar transport and are needed to maintain the morphology of the Golgi apparatus and the TGN (Uemura et al. 2014). Several other SNARE genes involved in post-Golgi membrane trafficking include the Qb-SNAREs *VTI11* and *12* and the Qc-SNAREs *SYP51* and *52* (De Benedictis et al. 2013). Transient expression of SYP51 and SYP52 in protoplasts revealed functional redundancy in vesicle sorting to the vacuole, localization to the TGN and endocytic compartments, and an inhibitory effect on fusion when accumulated on the tonoplast (De Benedictis et al. 2013).

In this study, we functionally characterized two *Arabidopsi*s genes, *BET11* and *BET12* (At3g58170 and At4g14455), believed to encode Qc-SNAREs (Uemura et al. 2004). These genes are Bet1/Sft1-like SNAREs that share 60 % aminoacid sequence identity with yeast *Sft1* gene, and when overexpressed are able to suppress the temperature-sensitive growth defect in *sft1*-*1* (Tai and Banfield 2001). A previous study determined that these two Arabidopsis genes (previously known as AtBS14a/b) are expressed in all plant tissues, including flowers, leaves, stem, roots and suspension cells (Uemura et al. 2004). We found that both genes were required for fertility, and that the N-terminal GFP fusion proteins localized to the Golgi apparatus, as observed in WT protoplasts. These results are in agreement with results by Uemura et al. (2004), who found that treatment of protoplasts with BFA, an inhibitor of Arf-GTPase guanine nucleotide exchange factors, caused accumulation of BET11-GFP and BET12-GFP at the Golgi. In that report the Golgi apparatus was labeled with marker Venus-SYP31, and colocalized well with BET11 and BET12 (Uemura et al. 2004). Our characterization of phenotypes in stable T-DNA double mutants suggested that the combined activity of *BET11* and *BET12* may be required for embryo development and proper pollen tube extrusion. Nonetheless, more work may be required to elucidate mechanistic and regulatory details related to the role the genes play at the Golgi apparatus in pollen tubes.

## Methods

### Molecular analysis of *BET11/12*

From a screen for potential gametophytic mutants, we chose the *Arabidopsis* *At3g58170* and *At4g14455* loci (*BET11* and *BET12*) for study (Additional file 1: Table S1). Then from a subset of 18 mutant lines corresponding to 12 SNARE genes, we chose the Columbia T-DNA lines SAIL_509_C09 (*bet11*-*1*) and SALK_124063 (*bet12*-*1*) for study. Genes were selected by high expression in *Arabidopsis* pollen as reported by GENEVESTIGATOR (https://www.genevestigator.com). The respective amino acid sequences were analyzed by T-Coffee, a consistency-based multiple sequence alignment program (Di Tommaso et al. 2011) and the on-line repositories UNIPROT (http://www.uniprot.org) and SWISS-MODEL (swissmodel.expasy.org). Plants from each line were genotyped with specific primer pairs for their corresponding T-DNA inserts and WT locus. Single homozygous lines did not show obvious vegetative or reproductive phenotypes, but homozygous/heterozygous (HM/HZ) double mutants for *bet11*-*1* and *bet12*-*1* (and vice versa) showed reduced seed set. No double HM mutants were recovered.

### Plant materials and growth conditions

The WT (ecotype Columbia) and T-DNA insertional lines, SAIL_509_C09 (*bet11*-*1*), and SALK_124063 (*bet12*-*1*) were obtained from the Arabidopsis Biological Resource Center (Columbus, OH, USA; http://abrc.osu.edu). Seeds were surface-sterilized in 30 % sodium hypochlorite and germinated on half-strength Murashige and Skoog medium without sucrose, then stratified at 4 °C for 96 h in the dark. Seedlings were grown at 21 °C under a 16-h photoperiod and 60 % relative humidity for approximately 5 days after the emergence of the radicle. Seedlings were then transferred to soil and genotyped with specific primer pairs for their corresponding T-DNA inserts and the WT locus. The primer sequences were for BET11-LP, 5′-GAGTAAGCCTGCCTCTGGTTC-3′, BET11-RP: 5′-TAGTACCCTGCCACGGTACAG-3′; BET12-LP: 5′-TCAAGCAAGCGGTTATGATTC-3′, BET12-RP: 5′-CACGAAAACTTACGCTTCTGG-3′, LB1:5′-GCCTTTTCAGAAATGGATAAATAGCCTTGCTTCC-3′, and LBb1.3: 5′-ATTTTGCCGATTTCGGA AC-3′. Complementation lines on the double mutant backgrounds were developed by transformation with the vector pZP221 carrying the native promoters and the full genomic sequence for either *BET11* or *BET12*, with a *GFP* tag on the N-terminus. Seedlings from the T_1_ generation were selected on agar based on resistance to antibiotic G418 and genotyped for the corresponding T-DNA insert and GFP sequence. A modified LP primer was used for complemented *bet12*-*1* lines. The sequence was as follows: BET12-modLP: 5′-CGGTTGGTTCACTAGTCTCT-3′.

### Quantitative PCR (qPCR)

Total RNA was extracted with the RNAeasy Plant Minikit (Qiagen, http://www.qiagen.com/) from WT, *bet11*-*1* and *bet12*-*1* seedlings. First-stranded cDNA was prepared from total RNA with the murine leukemia virus reverse transcriptase system (Promega, http://www.promega.com/). For quantitative PCR, a Power SYBR Green I Master Mix (Applied Biosystems, http://www.appliedbiosystems.com) was used with 150–200 nM primers, 20 ng/μL cDNA, and 50 μL reverse transcriptase reaction product. Reactions were run and analyzed on the AB 7500 Real Time PCR System (Applied Biosystems). Melting curve analyses and negative controls were used to exclude artifacts and low specificity. Reactions were performed in triplicate and averaged. Primers specific for the 3′ end of transcripts were designed by use of PRIMER EXPRESS 3.0 (Applied Biosystems). The primer sequences were for BET11, forward, 5′-CCATAGATCCAGGTGAATTCTGG-3′, and reverse, 5′-GCGGTTATGAGTATCGACCTCTTC-3′; BET12, forward, 5′-AGAGACTAGTGAACCAACCGA-3′, and reverse, 5′-ACAAAACTGCTAACATGAACCCA-3′; and ACT2, forward, 5′-GGCTCCTCTTAACCCAAAGGC-3′, and reverse, 5′-CACACCATCACCAGAATCCAGC-3′ as a normalization control.

### Analysis of subcellular localization of BET11 and BET12

Protoplasts prepared from leaves of 4-week-old Arabidopsis plants were co-transformed with *proBET11:BET11:GFP*, *proBET12:BET12:GFP*, Golgi marker construct *35S:Man1:GFP* and nuclear marker construct *35S:ERF4:mRFP* (not shown). Transformed protoplasts were observed by two-photon laser confocal microscopy and analyzed with use of Zeiss LSM Image Browser 3.5. *In planta* observations were not carried out.

### Genetic analyses

*BET11/12* double HM/HZ or HZ/HM mutants (*bet11*/−*bet12*/+, *bet11*/+*bet12*/−) were cross-pollinated with the WT to determine transmission efficiency of *bet11*-*1* and *bet12*-*1* alleles. The mutants were used as pollen receptors and donors. Transmission of the T-DNA alleles was analyzed by PCR with sampling of 100 progenies per cross with the WT and negative controls. Results were used to calculate the expected Mendelian segregation rate (E), the actual observed segregation rate (O) and the transmission efficiency rate (O/E × 100).

### Morphological characterization of *bet11*-*1* and *bet12*-*1* single and double mutants

Images of seed sets were recorded after dissection of at least 30 siliques from 7-week-old plants under a Lumar V12 fluorescence stereomicroscope (Zeiss, http://microscopy.zeiss.com/microscopy) connected to an AxioCam MRc5 CCD unit. In vitro pollen germination was performed according to Lin et al. (2014). Pollen was incubated in the dark for 4 h at 25 °C, then observed under a light microscope. Pollen was collected from at least three different plants, and 100 pollen grains were used to estimate pollen viability, with three replicates. Analysis of in vivo pollen-tube growth was as described (Szumlanski and Nielsen 2009; Lin et al. 2014). Emasculated pistils from (a) WT, (b) single homozygous *bet11*-*1* and *bet12*-*1*, (c) *bet11*/− *bet12*/+ and (d) *bet11*/+ *bet12*/− were cross-pollinated and collected after 48 h. Mature pollen morphology was analyzed by staining with 1.5 mg/mL 4′, 6-diamino-2-phenylindole (DAPI) (Vector Laboratories, http://www.vectorlabs.com) solution for 15 min in the dark, then observed under an Olympus BX51 epifluorescence microscope coupled to an Olympus DP70 CCD unit (Olympus, http://www.olympus-global.com/en/corc/company/lifescience).

## Results

### The mutant alleles *bet11*-*1* and *bet12*-*1* show distorted segregation rates

**Fig. 1:**
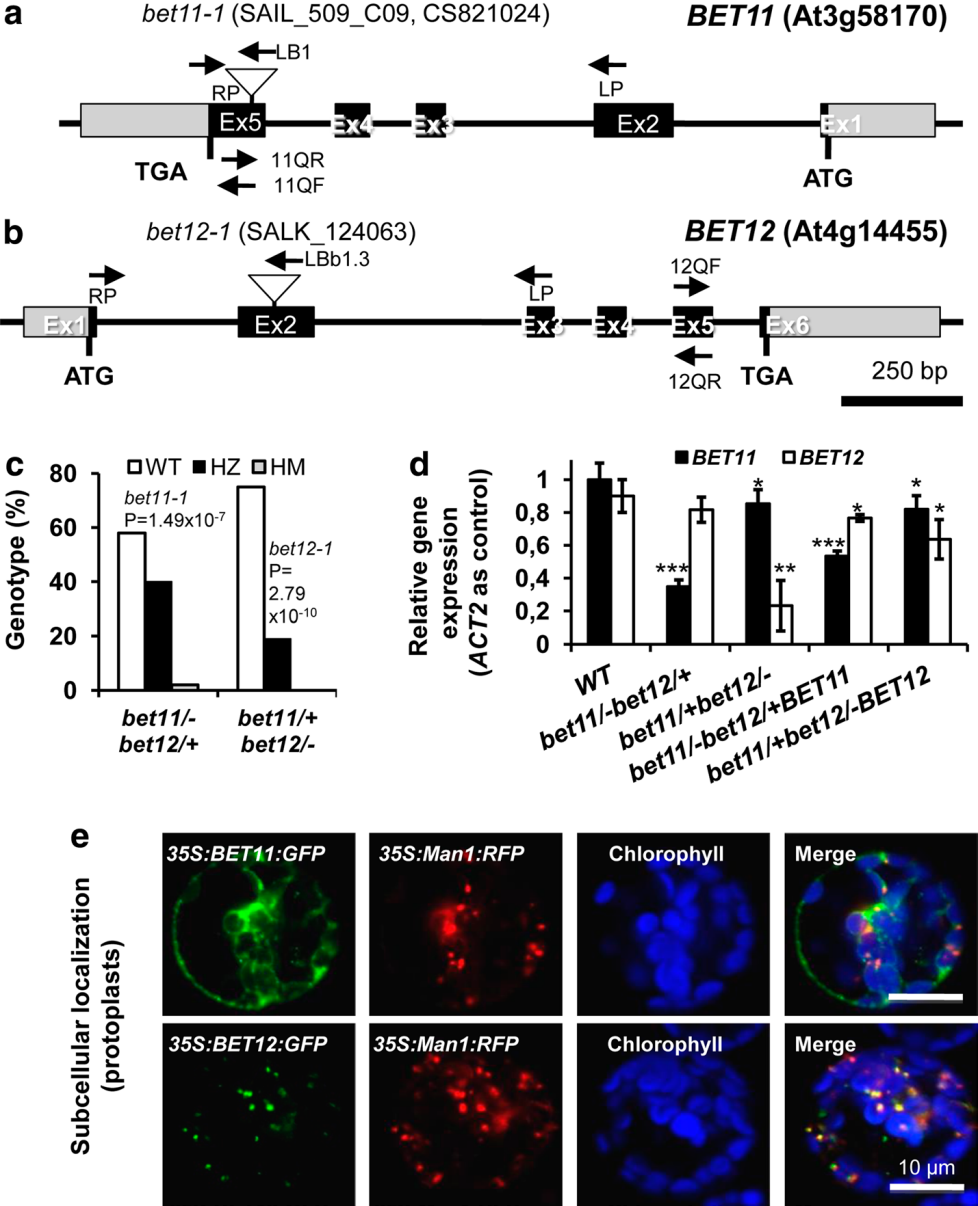
Molecular and cellular characterization of *Arabidopsis* *BET11* and *BET12* genes. **a** Diagram of *BET11* gene (At3g58170); exons appear as *black boxes*. The T-DNA insert in mutant line *bet11*-*1* (SAIL_509_C09) is on exon 5, the ATG start codon appears to the *right*, and the TGA stop codon is to the *left*. UTRs appear in *grey*. The location of the complementary primer sequences for genotyping and quantitative PCR appear as *arrowheads*. **b** Diagram of *BET12* gene (At4g14455). The T-DNA insert in mutant line *bet12*-*1* (SALK_124063) is on exon 2. **c** Segregation of *bet11*-*1* and *bet12*-*1* alleles in double mutants. Non-Mendelian segregation of the homozygous alleles is statistically significant, which suggests severe defects in transmission. **d** Quantification of relative expression of the 3′ end of *BET11* and *BET12* in the WT, double mutants and complementation lines. **e** Subcellular localization of BET11 and BET12 N-terminal, GFP-tagged proteins in *Arabidopsis* protoplasts. Both proteins showed partial colocalization with the Golgi marker Man1 (Man1:RFP). *Scale bars* 10 μm. Data are mean ± SD (*n* = 3) from three biological samples. *P < 0.1, **P < 0.01, ***P < 0.001 (Student *t* test) compared to the WT

A preliminary in silico analysis of the expression of several Arabidopsis fusogenic genes by Genevestigator (http://www.genevestigator.org) revealed that some were differentially expressed in reproductive tissues. *BET11* (At3g58170) showed expression in pollen, which suggested a role during plant reproduction, while no data was available for *BET12* (At4g14455). Both genes encode SNARE proteins of the Qc family, whose members feature a conserved glutamine residue within the coiled-coil SNARE motif (Uemura et al. 2004). T-DNA insertional mutants were available for each gene: line *bet11*-*1*, containing an insertion in exon 5 of *BET11* (Fig. 1a) and *bet12*-*1*, containing an insertion in exon 2 of *BET12* (Fig. 1a).

Our analyses of the phenotype of *bet11*-*1* and *bet12*-*1* single homozygous plants did not reveal any obvious defects in vegetative or reproductive growth. Moreover, PCR genotyping indicated that the mutants segregated in a 3:1 Mendelian ratio, which suggests that transmission of the T-DNA allele was more or less efficient. We could not isolate double homozygous *bet11/bet12* mutants. In fact, the corresponding homozygous/heterozygous mutants segregated in highly distorted, non-Mendelian ratios. In the *bet11*/−*bet12*/+ mutant (HM/HZ), the *bet11*-*1* T-DNA allele segregated with P = 1.49 × 10^−7^, and in the *bet11*/+ *bet12*/− mutant (HZ/HM), the *bet12*-*1* T-DNA allele segregated with P = 2.79 × 10^−10^ (Fig. 1c). These extremely significant values suggested reduced efficiency in the transmission of the T-DNA alleles caused by gametophyte or embryo lethality.

To better understand the reason for these defects, we analyzed the expression of the *BET11* and *BET12* genes in the WT and corresponding complementation lines, which carried the full genomic sequence driven by the native promoter. The expression of both genes was lower in the mutants than the WT (Fig. 1d). For instance, for homozygous *bet11*-*1*, the expression of *BET11* was reduced to 0.35 ± 0.04 (vs. 1.00 ± 0.10 in the WT) and 0.82 ± 0.09 in the heterozygous background, whereas for homozygous *bet12*-*1,* the expression of *BET12* was reduced to 0.23 ± 0.15 (vs. 0.90 ± 0.10 in the WT), and 0.77 ± 0.07 in the heterozygous background. For the complementation lines, the expression of both genes was increased to 0.54 ± 0.06 and 0.64 ± 0.12, respectively (Fig. 1d). Therefore, the *bet11*/− and *bet12*/− alleles appeared to be knockdown alleles that show reduced expression at the 3′ end.

### BET11 and BET12 proteins may reside at the Golgi apparatus

To better understand the function of the putative BET11 and BET12 proteins, we examined the localization patterns of their respective N-terminal, GFP-fusion proteins in *Arabidopsis* leaf protoplasts isolated from 4-week old *Arabidopsis* plants. Both GFP-tagged proteins showed some level of colocalization with the Golgi marker Man1 (Man1:RFP), especially BET12, which suggests that both proteins may reside at the Golgi apparatus (Fig. 1e). These results are in line with previous work (Uemura et al. 2012) and are in agreement with bioinformatic modeling of the function of both genes. For instance, alignment of amino acid sequences by use of T-Coffee suggested conservation between the putative Arabidopsis BET11 and BET12 proteins compared to the yeast homolog Sft1 (Additional file 2: Figure S1a). Results from the UNIPROT database indicated that both loci appear to encode canonical Qc-SNARE proteins that feature a SNARE coiled-coil domain, a transmembrane domain, a vesicular topological domain, and for BET12, a predicted phospho-serine site at residue 56 (Additional file 2: Figure S1b–c) that may regulate posttranslational dynamics. Moreover, SWISS-MODEL results suggest that each locus encodes a single chain able to assemble as a heterotetramer (Additional file 2: Figure S1b–c). This high level of similarity between both predicted proteins may indicate functional redundancy.

### Transmission of the homozygous mutant alleles is reduced in pollen

To better determine the extent of *BET11* and *BET12* function, we cross-pollinated *bet11*/*bet12* double mutants and the WT. Seeds were collected and progenies were genotyped by PCR to calculate the transmission efficiency of the *bet11*-*1* and *bet12*-*1* alleles (Table 1). For the *bet11* HM/*bet12* HZ line, maternal transmission of the heterozygous *bet12*-*1* allele showed distorted segregation of 21 % and a transmission efficiency of 42 %, which suggests defects in the maternal transmission of the allele. Even lower values in transmission of *bet12*-*1* occurred when the allele was transmitted paternally through pollen (22 %). For the *bet11* HZ/*bet12* HM line maternal transmission of the heterozygous *bet11*-*1* allele led to a segregation rate of 12 % and a transmission efficiency of 24 %. Paternal transmission of the heterozygous *bet11*-*1* allele led to a further reduction in the segregation rate, to 7 %, and a transmission efficiency of 14 %. Therefore, simultaneous reduction in activity of *BET11* and *BET12* genes may lead to impaired function of the male gametophyte and defects in embryo development.

**Table 1:** Transmission of the heterozygous *bet11*-*1* and *bet12*-*1* alleles is impaired in *bet11/12* double mutants

Cross	Segregating T-DNA allele	Expected segregation rate (E)	Observed segregation rate (O)	Transmission efficiency (O/E) × 100 (%)
*bet11* HM/*bet12* HZ (female) × WT (male)	*bet12*	50	21	42
WT (female) × *bet11* HM/*bet12* HZ	*bet12*	50	11	22
*bet12* HM/*bet11* HZ (female) × WT (male)	*bet11*	50	12	24
WT (female) × *bet12* HM/*bet11* HZ	*bet11*	50	7	14

Results show the segregation rates of the *bet11*-*1* and *bet12*-*1* alleles after reciprocal crosses involving the wild type (WT) and *bet11*/*bet12* double mutants in either the homozygous/heterozygous (HM/HZ) or the HZ/HM condition. From left to right, the table indicates the expected Mendelian segregation rate (E), the actual observed segregation rate (O) and the transmission efficiency (O/E × 100). For the *bet11* HM/*bet12* HZ line, maternal transmission efficiency of the *bet12*-*1* heterozygous (HZ) allele was low (42 %). Even lower values in the transmission efficiency of *bet12*-*1* allele were observed when the T-DNA allele was transmitted paternally through pollen (22 %). For the *bet11* HZ/*bet12* HM line, maternal and paternal transmission efficiencies of the heterozygous *bet11*-*1* allele were low (24 and 14 %). Data was collected from 100 segregating seedlings per cross

### *BET11* and *BET12* genes are required for fertilization

**Fig. 2:**
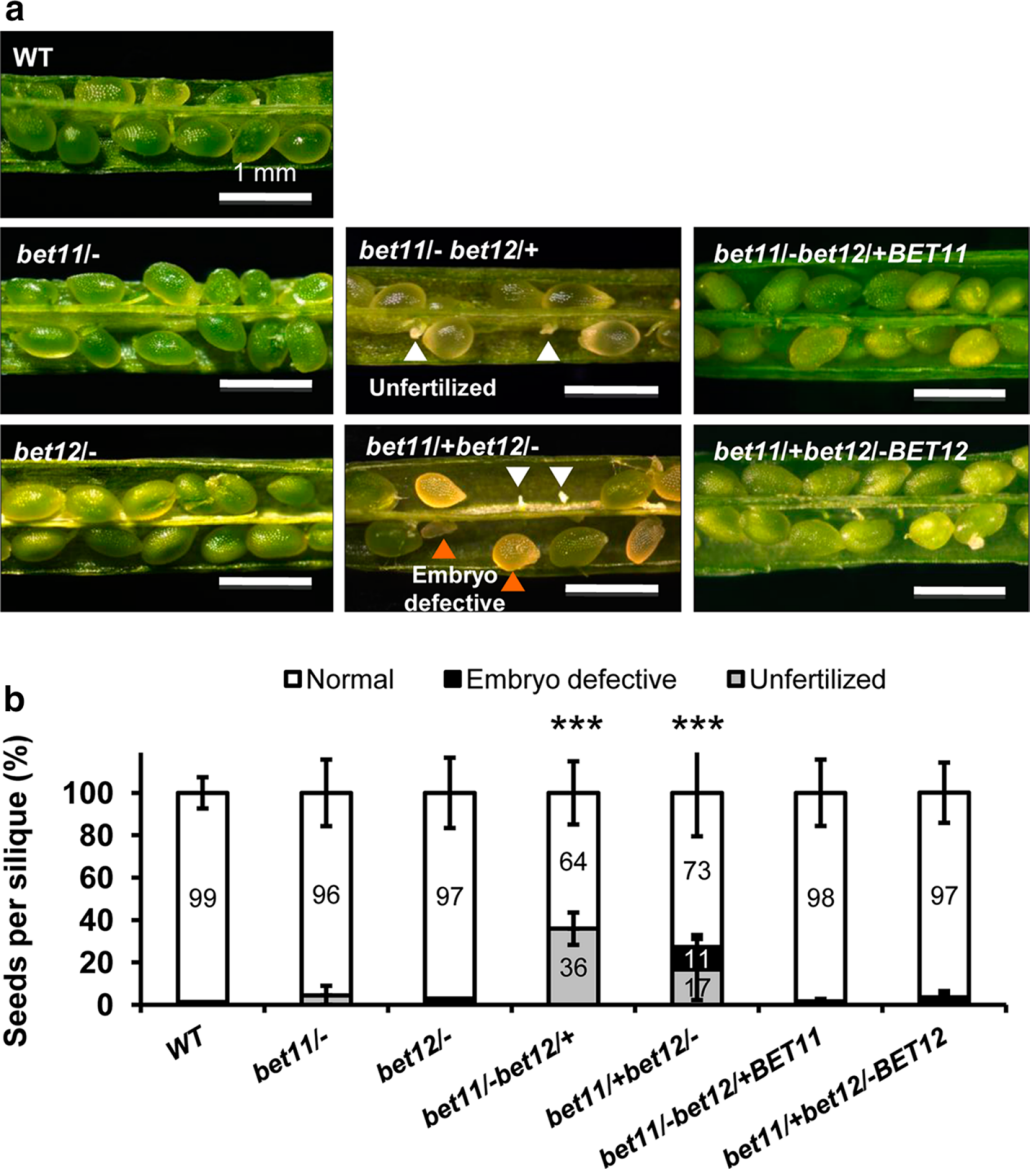
*Arabidopsis* *BET11* and *BET12* genes are required for fertility. **a** Seed development in the WT (*left*), single homozygous T-DNA lines (*left*), double mutants (homozygous/heterozygous) (*middle*) and complementation lines (*right*). *Scale bar* 1 mm. Data are mean ± SD (*n* = 3) from three biological samples. *P < 0.1, **P < 0.01, ***P < 0.001 (Student’s *t* test) compared with the WT

Analysis of seed development in mutant siliques from double mutants indicated that the *bet11*/−*bet12*/+ (HM/HZ) mutant showed significant failure to fertilize (white arrowheads, Fig. 2a), whereas the *bet11*/+ *bet12*/− (HZ/HM) mutant showed both fertilization failure and embryo developmental defects (white and orange arrowheads, Fig. 2a). The respective complementation lines (HM/HZ backgrounds) showed normal seed development. On analysis of seeds per silique, the WT and single mutants *bet11*/− and *bet12*/− had a mean of 47–43 seeds per silique, whereas the *bet11*/−*bet12*/+ and *bet11*/+ *bet12*/− double mutants had only about half the amount of seeds, 20–21 (P < 0.01 by Student’s *t* test). Moreover the *bet11*/+ *bet12*/− double mutant had a mean of 1–3 defective seeds per silique, so the *bet11*/− and *bet12*/− alleles may affect fertility differently, for instance *bet11*-*1*/− *bet12*/+ showed fertilization failure in 36 % of all samples (grey column segment, Fig. 1b), while *bet11*-*1*/+ *bet12*-*1*/− (HM) showed both embryo developmental defects and fertilization failure in 11 and 17 % of all samples, respectively (black column segment, Fig. 2b). These results strongly suggest that both *BET11* and *BET12* are required for fertility and embryo development.

### *BET11* and *BET12* genes are required for pollen tube elongation

**Fig. 3:**
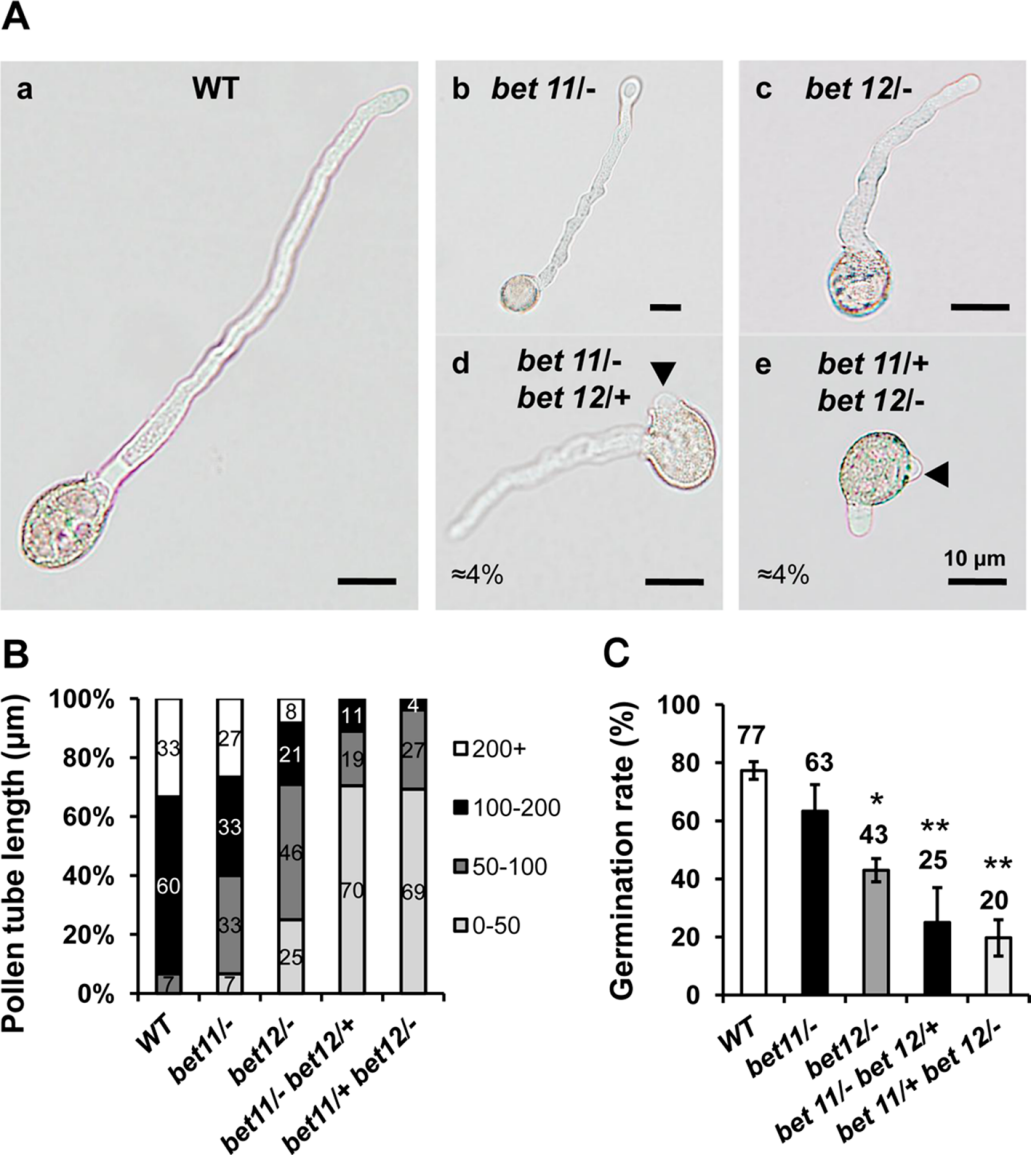
*BET11*/*12* mutants show extra pollen tubes and reduced pollen tube length in vitro. **A** Images from pollen tubes after germination. Shown are the WT *a*, single mutants *bet11*/− *b*, *bet12*/− *c*, and double mutants (HM/HZ) *bet11*/− *bet12*/+ *d*, and *bet11*/+ *bet12*/− *e*. **B** Quantification of pollen tube length (in μm). **C** Germination rate. Data are mean ± SD (*n* = 100) from three different biological samples. *P < 0.1, **P < 0.01, ***P < 0.001 (Student’s *t* test) compared to the WT. *Scale bars* 10 μm

In order to study pollen tube growth dynamics, pollen samples were first germinated in vitro and then were analyzed under the light microscope. In general, results from in vitro germination experiments showed that the single mutants exhibited reduced pollen tube length, and that this reduction became more severe in *bet11*/*bet12* double mutants (Fig. 3Aa–Ac); moreover, about 4 % of double mutant pollen grains extruded a secondary pollen tube (Fig. 3Ad–Ae). A tube of 100–200 μm long developed in about 60 % of WT pollen grains, and about 33 % grew tubes longer than 200 μm (Fig. 3B). However, in *bet11*-*1* and *bet12*-*1* single homozygous mutants, many of the pollen tubes (33–46 %) were 50–100 μm, followed by 100–200 μm (33–21 %), and <50 μm (7–25 %). The remainder were 100–200 μm. In the *bet11*/*bet12* double mutants (HM/HZ, and HZ/HM) no pollen tubes were >200 μm. The germination rate of the mutants was also different. For instance, in the WT about 77 % of pollen grains were able to germinate (Fig. 3C), but in *bet11*-*1* and *bet12*-*1* single homozygous mutants, this rate decreased to 63 and 43 %, respectively. In double mutants, the germination rate decreased significantly to 25 % in *bet11*/- *bet12*/+ and only 20 % in *bet11*/+ *bet12*/− (Fig. 3C). Thus, *BET11* and *BET12* activity may be crucial for proper pollen tube extrusion and elongation, and reduced activity of both genes may lead to reduced pollen germination and pollen tube length and formation of secondary pollen tubes.

Moreover, in vivo pollen germination experiments with WT pollen in single and double mutant *bet11*/*bet12* flowers did not reveal any apparent defects in the germination and elongation of pollen tubes within female tissues (Additional file 3: Figure S2a, upper panel). Use of the single mutants *bet11*/− and *bet12*/− as pollen donors and WT flowers as the female did not reveal any defects (Additional file 3: Figure S2a, lower panel). Nonetheless pollen from the *bet11*/−*bet12*/+ and *bet11*/+ *bet12*/− double mutants appeared to show poor germination and elongation; in fact, few pollen tubes were observed within female tissues after 48 h (Additional file 3: Figure S2a, lower panel, right side). Moreover, staining with DAPI indicated no significant differences in the morphology of mutant pollen grains; most grains (98–99 %) were tricellular and contained an identifiable vegetative cell and two sperm nuclei (Additional file 3: Figure S2b–c).

## Discussion

Plant reproduction starts with the deposition of pollen grains onto the stigma of the pistil. After the grains germinate, the pollen tubes grow through the transmitting tract of the style and septum to reach the embryo sac, where double fertilization takes place (Kaya et al. 2014). Pollen tubes expand by tip growth, whereby the apex of the cell grows much faster than the other sides, thereby generating an elongated structure (Kaya et al. 2014). In this study we show that two putative, highly similar Qc-SNARE genes, *BET11* and *BET12*, are involved in pollen tube growth and that double mutants in a homozygous/heterozygous condition show reduced transmission of the heterozygous alleles (Table 1), defects in fertilization and embryo development (Fig. 2), reduced pollen tube elongation and the extrusion of secondary pollen tubes (Fig. 3), which suggests possible loss of polarity. The respective N-terminal GFP-tagged proteins mostly localized to the Golgi in protoplasts, so they may play a role during endomembrane trafficking (Fig. 1).

The SNAREs and other fusogenic determinants have important roles during pollen tube development. For instance, development of secondary pollen tubes (as in *bet11/bet12* mutants) has been linked to reduced expression of the *PIP5K4* gene, which encodes the phosphatidylinositol-4-monophosphate 5-kinase 4, an enzyme in charge of the conversion of phosphatidylinositol (4,5)-bisphosphate (or PtdIns(4,5)P_2_). This molecule plays key roles in actin dynamics, vesicle trafficking, and ion transport (Sousa et al. 2008). Lily pollen tubes overexpressing the actin-binding protein (ABP) LILIM1 showed protrusion of multiple tubes from one pollen grain and aggregation of FM4-64-labeled compartments, which suggests possible cross-talk between endomembrane trafficking and cytoskeletal organization (Wang et al. 2008). In fact, Rho GTPases such as ROP1 and interacting protein RIC3 are believed to function as spatial regulators of ABPs including F-actin, profilin and formin and thus help determine growth polarity (Chebli et al. 2013; Cheung et al. 2010).

Within the Ras superfamily of proteins, Rab GTPases are thought to be required for selective vesicle attachment to target vesicles (Carr and Rizo 2010). When bound to GDP, RABs are inactive, but when bound to GTP, they become activated and interact with several effector proteins (Ung et al. 2013), including SNAREs, during the tethering of cargo at post-Golgi compartments (Cai et al. 2007). In pollen tubes, loss of Arabidopsis *RABA4D* expression led to disrupted polar growth and changes in cell wall patterning and compromised in vivo pollen tube growth and guidance (Szumlanski and Nielsen 2009). Conversely, overexpression of C-terminal–truncated versions of the Rho guanine exchange factor AtRopGEF12 also caused disturbed pollen tube growth, including isotropic growth (Zhang and McCormick 2007).

Moreover systems biology model based on the behavior of *Arabidopsis* RABA4D required the activity of SNAREs for vesicle fusion and GTPases for budding (Kato et al. 2010). In this model, specific GTPases favor budding from specific compartments at specific rates, whereas the rate of vesicle fusion depends on the rate of SNARE pairing between the vesicle and the target (Kato et al. 2010). However it is not known whether this model applies to *bet11/bet12* mutants.

Recent work has shown that the *Arabidopsis* Qc-SNARE SYP61 and Qa-SNARE SYP121 are required for coordinated targeting to the plasma membrane of aquaporin PIP2;7, a process that requires anteroretrogade passage through the TGN during secretion or during retrograde uptake from the plasma membrane (Hachez et al. 2014). This process also appears to mediate expansion of root hairs (Hachez et al. 2014), which are cells well known for showing SNARE-dependent polarized tip growth (Larson et al. 2014).

In *Arabidopsis*, defects in the expression of SNARE genes induce collapse of Golgi compartments into the endoplasmic reticulum (ER) (Chatre et al. 2005). A similar phenomenon was reported for the R-SNARE gene *SEC22* in *Arabidopsis* mutants (El-Kasmi et al. 2011), which show fragmentation of Golgi stacks, presumably because of blocked membrane fusion during anterograde or retrograde ER to Golgi trafficking (El-Kasmi et al. 2011). A similar process might occur in *bet11*/*bet12* double mutant pollen tubes. Nonetheless, examination of endomembrane compartments by transmission electron microscopy is required to address this question and help determine whether *bet11/bet12* mutants are required for maintenance of proper structure of the Golgi or TGN in pollen tubes.

Protein-driven membrane fusion events are essential in all organisms (Pérez-Vargas et al. 2014). They are crucial to achieve intracellular trafficking, neurotransmitter secretion, cell mating and fertilization. They are also key to the development of tissues and organs in multicellular organisms (Pérez-Vargas et al. 2014).

In this study, we characterized the role of two putative *Arabidopsis* Qc-SNARE genes, *BET11* and *BET12*, in plant growth. Results from the characterization of T-DNA double mutants strongly suggest that these two genes perform largely overlapping functions during fertilization. Both are required during polarized extrusion of pollen tubes, as evidenced by reduced pollen tube length and the development of secondary pollen tubes (Table 1). Genetic data also suggests a role for both genes during embryo development, especially for *BET12* (Table 1). These roles seem to be a departure from the role that bet11p plays in yeast, in which the *bet1*-*1* allele leads only to minor growth defects as compared with the WT (Rogers et al. 2013). This difference in behavior could be interpreted as an evolutionary gain of function for BET Qc-SNAREs in multicellular eukaryotes. If so, *BET11/BET12* homologs in plants and metazoans may have a specialized role during polarized growth and embryo development.

In conclusion, *Arabidopsis* *BET11* and *BET12* genes are required for polarized growth in pollen and for embryo development. Nonetheless, more work is required to identify the role of the BET11 and BET12 in anterograde and retrograde traffic, whether fragmentation of the Golgi apparatus occurs in *bet11*/*bet12* mutants, and to determine the type of cargo specified by BET11 and BET12 in vesicles. We believe that this study may pave the way for further characterization of the function played by these two genes during reproduction and uncover interactions unknown at the present time.

## Additional files

Additional file 1: **Table S1.** Genetic screen of suspected fusogenic factors. Alleles were selected based on expression levels in silico, possible ER/Golgi localization and the availability of T-DNA lines. Double mutants were synthesized only for alleles corresponding to *BET11/12* and *SFT11/12*. The *SFT11/12* double mutant remains uncharacterized.

Additional file 2: **Figure S1.** The Arabidopsis Qc-SNARE genes *BET11* and *BET12* encode membrane integral proteins. **a** Alignment of amino acid sequences with the consistency-based T-Coffee program (http://www.tcoffee.org) revealed structural conservation (67.1 %) between the Arabidopsis BET11 and BET12 proteins and the yeast homolog Sft1. The residue color scheme shows the primary library support for the alignment of the considered residue on a scale from 0 (blue, poorly supported) to 9 (dark red, strongly supported). **b** and **c** left, diagram of the structural domains of BET11 and BET12 proteins adapted from UNIPROT (http://www.uniprot.org). Both proteins feature an N-terminal SNARE coiled-coil domain (red), a C-terminal transmembrane domain (red), and a C-terminal vesicular topological domain (green); however, only BET11 features a phospho-serine at residue 56. Right, 3D structures according to the SWISS-MODEL repository (swissmodel.expasy.org). Both BET11 and BET12 are believed to assemble as single chains that may associate as heterotetramers. Symbols: * = identity match,: = high structural homology,. = high similarity.

Additional file 3: **Figure S2.** Pollen grains from *BET11*/*12* mutants show reduced pollen tube growth in vivo but no developmental defects before germination. **a** Top row, confocal microscopy of cross of *bet11/12* single and double mutants used as pollen donors (♂) with the WT as the receptor (♀). Bottom row, the WT was used as a pollen receptor (♀), with *bet11/12* single and double mutants as pollen donors. At least 3 flowers from 3 different plants were analyzed per line. **b** DAPI staining of pollen grains by confocal microscopy. **c** Quantification of tricellular pollen abundance in the WT, single *bet11/12* mutants and *bet11/bet12* double mutants. No statistically significant differences were observed. Data are mean ± SD (*n* = 100) from three different biological samples.
