# Supplementary material for: Arabidopsis Qc-SNARE genes BET11 and BET12 are required for fertility and pollen tube elongation
Source: Bot Stud. 2015 Sep 2;56:21. doi: 10.1186/s40529-015-0102-x (PMC5430320; doi:10.1186/s40529-015-0102-x)
Supplement: Supplementary file 1 — Table S1. Genetic screen of suspected fusogenic factors. Alleles were selected based on expression levels in silico, possible ER/Golgi localization and the availability of T-DNA lines. Double mutants were synthesized only for alleles corresponding to BET11/12 and SFT11/12. The SFT11/12 double mutant remains uncharacterized. [file 40529_2015_102_MOESM1_ESM.doc]

| **Yeast gene** | ***Arabidopsis* homolog** | **Expression level in pollen**  **(GENEVESTI-**  **GATOR)** | **Name** | **Gene function** | **Lines** | **Location**  **of T-DNA** | **Availability** | **Observed segregation rate** |
| --- | --- | --- | --- | --- | --- | --- | --- | --- |
| ***KAR5*** | *At4g24610* | Low |  | Spindle associated | SAIL_125G09  CS806137 | Intron (HZ) | Yes | Line not ordered |
| ***KAR5*** | *At4g24610* | Low |  | Unknown protein,  plasma membrane | SAIL_270B07 CS812530 | Exon (HZ) | Yes | Line not ordered |
| ***KAR5*** | *At4g27595* | N |  | Unknown protein, chloroplast | SAIL_222A09 CS810266 | Exon (HZ) | Yes | Line not ordered |
| ***KAR8*** | *At2g35720* | Low | ~~-~~ | DnaJ | SAIL_199H05 CS872063 | Intron (HZ) | Yes | Line not ordered |
| ***KAR8*** | *At2g35720* | Low | ~~-~~ | DnaJ | SAIL_110H09 CS871209 | Exon (HZ) | Yes | Line not ordered |
| ***KAR8*** | *At2g35720* | Low | ~~-~~ | DnaJ | SAIL_110G10 CS805393 | Exon (HZ) | Yes | Line not ordered |
| ***KAR8*** | *At2g35720* | Low | ~~-~~ | DnaJ | SAIL_656B03 CS828505 | Exon (HZ) | Yes | Line not ordered |
| ***PRM3*** | *At1g50720* | Low | Stig1 | Recruitment of SNAREs | SM_40522, John Innes Centre, transposon line  (CS127233) | Exon (?) | Yes | Line not ordered |
| ***PRM3*** | *At1g50720* | Low | Stig1 | Unknown | SM.40534, John Innes Centre, transposon line  (CS127245) | Exon(?) | Yes | Line not ordered |
| ***PRM2*** | *At1g73290* | Mid to  high | *SCPL5* | Endomembranes, role in proteolysis | SAIL_175A07  CS808469 | Intron  (HZ) | Yes | All WT |
| ***PRM2*** | *At1g73290* | Mid to  high | *SCPL5* | Endomembranes, role in proteolysis | SAIL_570B10 CS824253 | Exon  (HZ) | Yes | Line not ordered |
| ***SEC63*** | *At4g21180* | Low | *AtERdj2B* | DnaJ (type III) | SALK_117783 | Exon (HZ) | Yes | Line not ordered |
| ***SEC63*** | *At4g21180* | Low | *AtERdj2B* | DnaJ (type III) | SALK_007095 | Exon (HZ) | Yes | Line not ordered |
| ***SEC72/KAR7*** | *At4g11260* | Low | *SGT1B* | SCF ubiquitin ligase complex | SAIL_1212E06  CS878518 | Exon (HZ) | Yes | Line not ordered |
| ***SEC72/KAR7*** | *At4g11260* | Low | *SGT1B* | SCF ubiquitin ligase complex | SAIL_828A10  CS877156 | Intron  (HZ) | Yes | Line not ordered |
|  | *At2g46170* | High | *RTNLB3* | ER protein | RATM11-1029-1_H (Ds transposon line,flanking sites confirmed) | Exon | RARGE  (RIKEN) | Line not ordered |
|  | *At1g18700* | Middle | - | DnaJ at ER | SAIL_916C03  CS841271 | Intron  (HZ) | Yes | WT 3: HZ 12: HM 8 |
|  | *At1g18700* | Middle | - | DnaJ at ER | WISCDSLOXHS050_01E  CS904709 | Intron | Yes | Line not ordered |
|  | *At1g18700* | Middle | - | DnaJ at ER | WISCDSLOXHS067_04E  CS906365 | Exon | Yes | Line not ordered |
|  | *At1g18700* | Middle | - | DnaJ at ER | WISCDSLOXHS071_04E CS906749 | Exon | Yes | Line not ordered |
|  | *At4g37910* | Low | - | Mitochondrial  Hsp70 | SALK_081385 | Intron  (HZ) | Yes | Line not ordered |
|  | *At4g37910* | Low | - | Mitochondrial  Hsp70 | SALK_081383 | Intron  (HZ) | Yes | Line not ordered |
|  | *At4g10250* | Low |  | ER Hsp20 | RATM13-4798-1_G  (Ds transposon line,flanking sites confirmed) | Exon | RARGE | Line not ordered |
|  | *At4g25200* | Low | *AtHsp23.6-mito* | Mitochondrial small heat shock protein | SAIL_373_B09  CS817244 | Exon  (HZ) | Yes | Line not ordered |
| ***PAM18*** | *At5g03030* | Low |  | DnaJ | SALK_091892 | Intron  (HZ) | Yes | Line not ordered |
| ***PAM18*** | *At5g03030* | Low |  | DnaJ | SALK_091890 | Intron  (HZ) | Yes | Line not ordered |
| ***PAM18*** | *At5g03030* | Low |  | DnaJ | SALK_091883 | Exon  (HZ) | Yes | Line not ordered |
| ***PAM18*** | *At5g03030* | Low |  | DnaJ | SALK_091873 | Exon  (HZ) | Yes | Line not ordered |
| ***JID1*** | *At3g08910* | High |  | DnaJ | SAIL_37_B04  CS870416 | Intron  (HZ) | Yes | All WT |
| ***JID1*** | *At2g41520* | Low |  | DnaJ-like | SAIL_36_C08 CS801731 | Intron  (HZ) | Yes | Line not ordered |
| ***JID1*** | *At1g59725* | Low |  | DnaJ | SALK_004406 | Exon  (HZ) | Yes | Line not ordered |
| ***JID1*** | *At1g59725* | Low |  | DnaJ | SALK_041529 | Exon  (HZ) | Yes | Line not ordered |
| ***JID1*** | *At1g59725* | Low |  | DnaJ | SALK_004406 | Exon  (HZ) | Yes | Line not ordered |
| ***Sec18p/NSF*** | *At3g16290* | Low | *EMB2083* | ATPase activity,  chloroplast envelope | SALK_082501 | Intron  (HZ) | Yes | Line not ordered |
| ***Sec18p/NSF*** | *At3g16290* | Low | *EMB2083* | ATPase activity,  chloroplast envelope | SAIL_207G07 CS809696 | Exon (HZ) | Yes | Line not ordered |
| ***Sec18p/NSF*** | *At3g16290* | Low | *EMB2083* | ATPase activity,  chloroplast envelope | SAIL_520G02 CS822062 | Exon  (HZ) | Yes | Line not ordered |
| ***Sec18p/NSF*** | *At1g79560* | Low | *EMB1047* | ATPase activity,  chloroplast, mitochondrion, plastid | SALK_049724 | Intron  (HZ) | Yes | Line not ordered |
| ***Sec18p/NSF*** | *At3g04340* | Low | *EMB2458* | ATPase activity,  chloroplast, chloroplast envelope | RATM52-0250-1_G  (Ds transposon line,flanking sites confirmed) | Intron | Yes | Line not ordered |
| ***Sec18p/NSF*** | *At3g04340* | Low | *EMB2458* | ATPase activity,  chloroplast, chloroplast envelope | RAFL06-68-L19  T-DNA activation line | Exon | Yes | Line not ordered |
| ***Sec18p/NSF*** | *At1g21690* | High | *EMB1968* | ATPase activity  nucleolus | SALK_049715 | Intron  (HZ) | Yes | Line not ordered |
| ***Sec18p/NSF*** | *At1g21690* | High | *EMB1968* | ATPase activity  nucleolus | SALK_049714 | Exon  (HZ) | Yes | Line not ordered |
| ***PRM5*** | *At2g17140* | High | *F6P23.26* | Contains pentatricopeptide repeat | SALK_026567 | Exon  (HZ) | Yes | Line not ordered |
| **Qa SNARES**  **SYP81 fam** | *At1g51740* | High | *SYP81* | ER | None  available | - | No | Line not ordered |
| **Qb**  **SNARES**  **MEMB**  **11 fam** | *At2g36900* | Middle | *MEMB11* | ER | GABI_055E08  (insertion confirmed)  NASC ID: N405240 | Intron | Yes | Line not ordered |
| **Qb**  **SNARES**  **MEMB**  **11 fam** | *At2g36900* | Middle | *MEMB11* | ER | WiscDsLox493G07  CS858509 | Intron (HZ) | Yes | Line not ordered |
| **Qb**  **SNARES**  ***SEC20 fam*** | *At3g24315* | Low | *SEC20* | ER | SALK_040675C | Exon  (HM) | Yes | Line not ordered |
| **Qc SNARES**  **BET/STF**  **fam** | *At3g58170* | Low | *BET11* | ER | SALK_150639C | Intron  (HM) | Yes | All WT |
| **Qc SNARES**  **BET/STF**  **fam** | *At3g58170* | Low | *BET11* | ER | SAIL_501C09 CS821024 | Exon  (HZ) | Yes | WT 5 : HM 11 : HZ 11 |
| **Qc SNARES**  **BET/STF**  **fam** | *At4g14455* | Not available | *BET12* | ER-Golgi | SALK_124063 | Exon  (HZ) | Yes | WT 3 : HM 7 : HZ 7 |
| **Qc SNARES**  **BET/STF**  **fam** | *At4g14600* | Low in sperm | *SFT11* | Unknown | SALK_065900C | Intron  (HM) | Yes | WT 4 : HM 3 : HZ 5 |
| **Qc SNARES**  **BET/STF**  **fam** | *At4g14600* | Low in sperm | *SFT11* | Unknown | SALK_090509 | Exon  (HZ) | Yes | WT 14 : HZ 2 |
| **Qc SNARES**  **BET/STF**  **fam** | *At1g29060* | Low in sperm | *SFT12* | Unknown | SALK_054695C | Exon  (HM) | Yes | All HM |
| **Qc SNARES**  **USE fam** | *At3g55600* | High in sperm | *USE12* | ER | None  available | - | No |  |
| **Qc SNARES**  **USE fam** | *At1g54110* | Low in sperm | *USE11* | ER | None  available | - | No |  |
| **Qc SNARES**  **SYP fam** | *At3g45280* | High in sperm | *SYP72* | SNARE, located at the ER | SALK_045561C | Intron  (HM) | Yes | WT 2 : HM 4 |
| **Qc SNARES**  **SYP fam** | *At3g45280* | High in sperm | *SYP72* | SNARE, located at the ER | SALK_005228 | Exon  (HZ) | Yes | WT 12 : HZ 1 |
| **Qc SNARES SYP fam** | *At3g61450* | High in sperm | *SYP73* | SNARE, located at the ER | SALK_091845C | Exon  (HM) | Yes | All WT |
| **Qc SNARES SYP fam** | *At3g61450* | High in sperm | *SYP73* | SNARE, located at the ER | SALK_090721 | Exon  (HZ) | Yes | WT 3 : HM 4 : HZ 1 |
| **Qc SNARES SYP fam** | *At3g61450* | High in sperm | *SYP73* | SNARE, located at the ER | SALK_134629 | Exon  (HM) | No | All WT |
| **R**  **SNARES**  **SEC22 fam** | *At1g11890* | Low in sperm | *SEC221* | SNARE, located at the ER | SAIL_250_F12 CS811678 | Exon  (HZ) | No | WT 10 : HM 1 : HZ 16 |
| **R**  **SNARES**  **SEC22 fam** | *At1g11890* | Low in sperm | *SEC221* | SNARE, located at the ER | SAIL_736_F03 CS832909 | Exon  (HZ) | Yes | All WT |
| **R**  **SNARES**  **SEC22 fam** | *At1g11890* | Low in sperm | *SEC221* | SNARE, located at the ER | SALK_042619 | Exon HZ  5’-UTR | Yes | All WT |
| **R**  **SNARES**  **SEC22 fam** | *At5g52270* | Low in sperm | *SEC222* | SNARE, located at the ER | SALK_072000 | Exon  (HM) | Yes | WT 10 : HM 12 : HZ 5 |
| **SM regulator** | *At2g17980* | *NA* | *SLY11* | - | None available | - | No |  |
| **SNAP regulator** | *At4g20410* | Mid in sperm | *γ-SNAP* | SNARE regulator at ER | SAIL_409C09 CS879032 | Intron  (HZ) | Yes | WT 1 : HM 22 |
| **SNAP regulator** | *At4g20410* | Mid in sperm | *γ-SNAP* | SNARE regulator at ER | SALK_150198 | Exon  (HZ) | Yes | WT 3 : HM 5 : NA 9 |
