# Supplementary figures and images for: Arabidopsis Qc-SNARE genes BET11 and BET12 are required for fertility and pollen tube elongation
Source: Bot Stud. 2015 Sep 2;56:21. doi: 10.1186/s40529-015-0102-x (PMC5430320; doi:10.1186/s40529-015-0102-x)

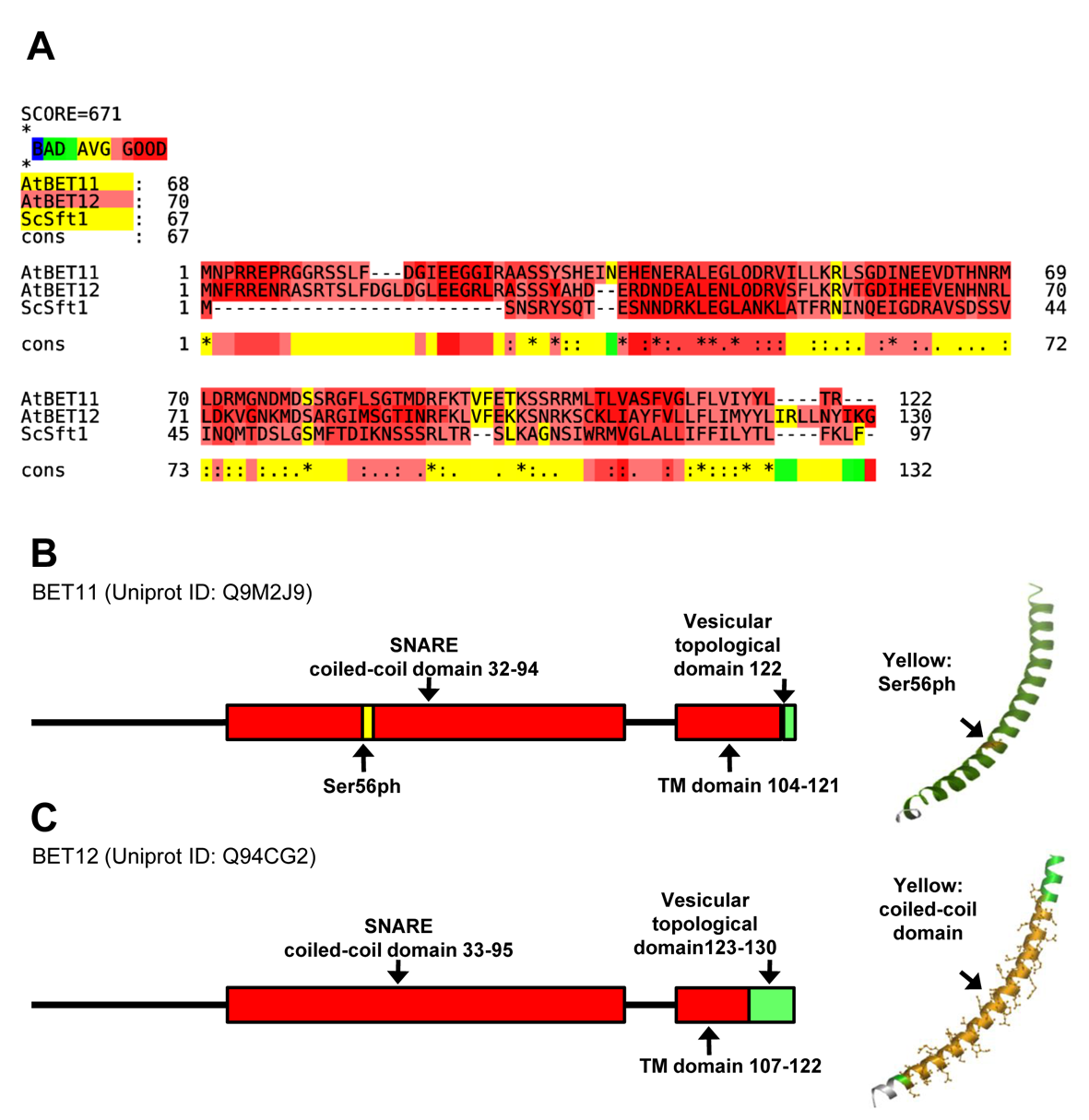

Supplement: Supplementary file 2 — Figure S1. The Arabidopsis Qc-SNARE genes BET11 and BET12 encode membrane integral proteins. a Alignment of amino acid sequences with the consistency-based T-Coffee program (http://www.tcoffee.org) revealed structural conservation (67.1 %) between the Arabidopsis BET11 and BET12 proteins and the yeast homolog Sft1. The residue color scheme shows the primary library support for the alignment of the considered residue on a scale from 0 (blue, poorly supported) to 9 (dark red, strongly supported). b and c left, diagram of the structural domains of BET11 and BET12 proteins adapted from UNIPROT (http://www.uniprot.org). Both proteins feature an N-terminal SNARE coiled-coil domain (red), a C-terminal transmembrane domain (red), and a C-terminal vesicular topological domain (green); however, only BET11 features a phospho-serine at residue 56. Right, 3D structures according to the SWISS-MODEL repository (swissmodel.expasy.org). Both BET11 and BET12 are believed to assemble as single chains that may associate as heterotetramers. Symbols: * = identity match,: = high structural homology,. = high similarity. [file 40529_2015_102_MOESM2_ESM.tif]

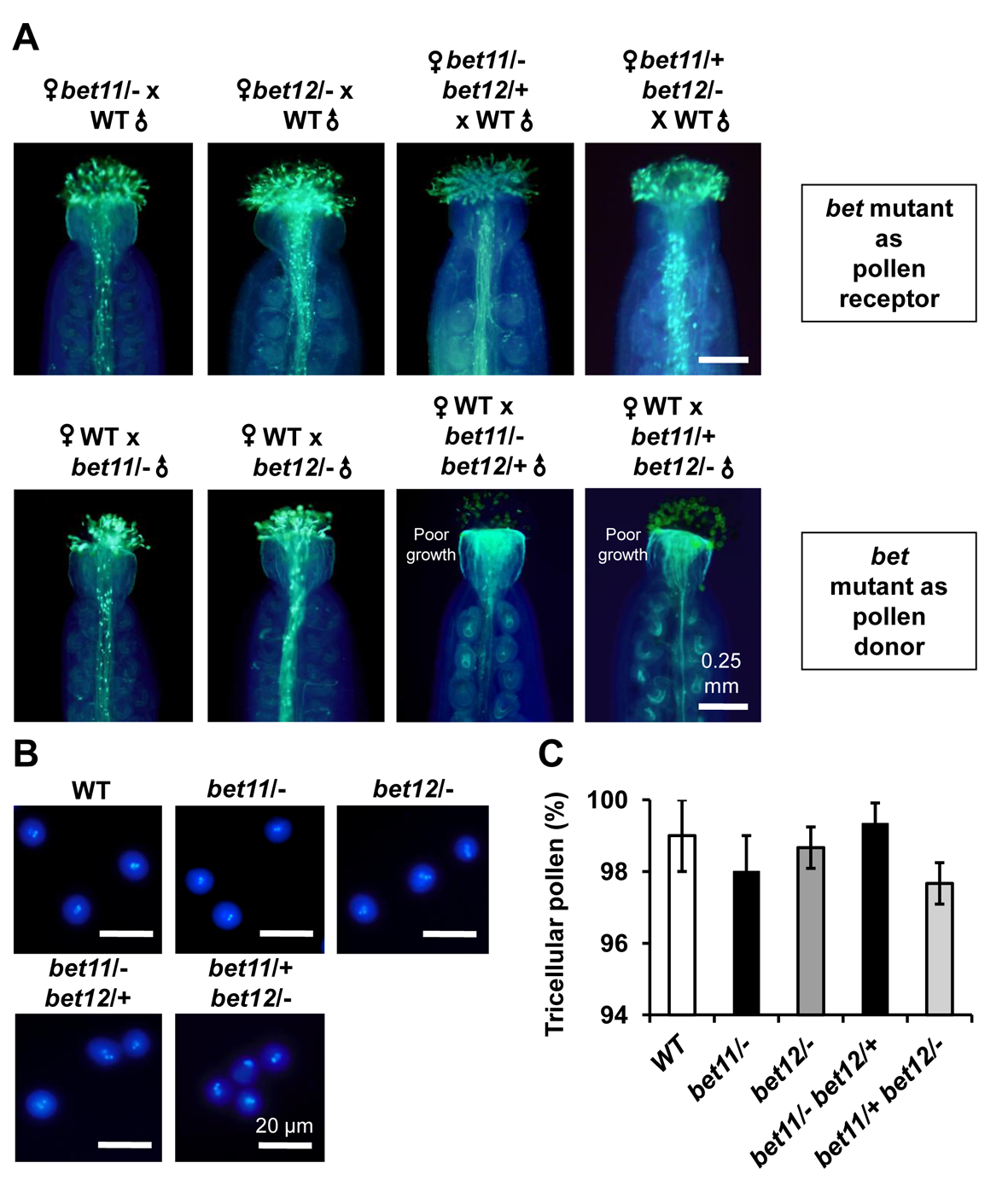

Supplement: Supplementary file 3 — Figure S2. Pollen grains from BET11/12 mutants show reduced pollen tube growth in vivo but no developmental defects before germination. a Top row, confocal microscopy of cross of bet11/12 single and double mutants used as pollen donors (♂) with the WT as the receptor (♀). Bottom row, the WT was used as a pollen receptor (♀), with bet11/12 single and double mutants as pollen donors. At least 3 flowers from 3 different plants were analyzed per line. b DAPI staining of pollen grains by confocal microscopy. c Quantification of tricellular pollen abundance in the WT, single bet11/12 mutants and bet11/bet12 double mutants. No statistically significant differences were observed. Data are mean ± SD (n = 100) from three different biological samples. [file 40529_2015_102_MOESM3_ESM.tif]
